# Roles of Long Non-coding RNAs in the Development of Chronic Pain

**DOI:** 10.3389/fnmol.2021.760964

**Published:** 2021-11-23

**Authors:** Zheng Li, Xiongjuan Li, Wenling Jian, Qingsheng Xue, Zhiheng Liu

**Affiliations:** ^1^Department of Anesthesiology, The First Affiliated Hospital of Shenzhen University, Shenzhen Second People’s Hospital, Shenzhen, China; ^2^Department of Geriatric & Spinal Pain Multi-Department Treatment, The First Affiliated Hospital of Shenzhen University, Shenzhen Second People’s Hospital, Shenzhen, China; ^3^Department of Anesthesiology, Ruijin Hospital, School of Medicine, Shanghai Jiao Tong University, Shanghai, China

**Keywords:** long non-coding RNA, chronic neuropathic pain, chronic cancer-related pain, dorsal root ganglion, spinal cord

## Abstract

Chronic pain, a severe public health issue, affects the quality of life of patients and results in a major socioeconomic burden. Only limited drug treatments for chronic pain are available, and they have insufficient efficacy. Recent studies have found that the expression of long non-coding RNAs (lncRNAs) is dysregulated in various chronic pain models, including chronic neuropathic pain, chronic inflammatory pain, and chronic cancer-related pain. Studies have also explored the effect of these dysregulated lncRNAs on the activation of microRNAs, inflammatory cytokines, and so on. These mechanisms have been widely demonstrated to play a critical role in the development of chronic pain. The findings of these studies indicate the significant roles of dysregulated lncRNAs in chronic pain in the dorsal root ganglion and spinal cord, following peripheral or central nerve lesions. This review summarizes the mechanism underlying the abnormal expression of lncRNAs in the development of chronic pain induced by peripheral nerve injury, diabetic neuropathy, inflammatory response, trigeminal neuralgia, spinal cord injury, cancer metastasis, and other conditions. Understanding the effect of lncRNAs may provide a novel insight that targeting lncRNAs could be a potential candidate for therapeutic intervention in chronic pain.

## Introduction

Chronic pain is an extremely prevalent healthcare issue that affects the quality of life of patients, resulting in an annual financial impact (van Hecke et al., 2014; Hamood et al., 2018). It can be generally categorized as chronic cancer-related pain or chronic non-cancer-related pain, such as chronic neuropathic pain (CNP) and chronic postsurgical or posttraumatic pain (Treede et al., 2019). Although many studies have elucidated the mechanisms underlying the development of chronic pain, only a few currently available clinical therapeutic strategies effectively alleviate pain symptoms in patients with limited unwanted side effects. Thus, it is imperative to explore novel targets for the treatment of chronic pain.

Long non-coding RNA, which consists of more than 200 nucleotides, is a non-coding RNA that lacks a complete open reading frame (Batista and Chang, 2013). Although they cannot translate into detectable proteins individually, long non-coding RNAs (lncRNAs) can play a crucial role in the expression and translation of other genes and whole gene networks by interacting with DNA, proteins, and other RNAs (Wang and Chang, 2011). Accumulating evidence indicates that lncRNAs are potent regulators of physiological and pathological processes, such as embryonic development, cancer, inflammation, and neurological diseases (Ulitsky and Bartel, 2013). Recently, many studies have identified changes in the expression and important role of lncRNAs in chronic pain models. Therefore, this review aimed to explore the roles and mechanisms of lncRNAs in the development of chronic pain, including CNP and chronic cancer-related pain (CCRP).

## The Role of lncRNAs in the Nervous System and the Pain-Signaling Pathway

Dysregulated lncRNA expression has been found in damaged nerves, primary sensory dorsal root ganglion neurons, spinal cord, and postsynaptic dorsal horn after peripheral nerve lesions or spinal cord injury (SCI). Under these conditions, accumulating evidence has shown the effect of the interaction between lncRNAs and miRNAs in the development of chronic pain. As a competitive endogenous RNA (ceRNA) (Chen et al., 2017; Sun et al., 2019), lncRNAs can competitively bind miRNAs, inhibit the interaction between miRNAs and downstream genes, and regulate the transcription and expression of downstream genes. For example, lncRNA MALAT1 can sponge miR-129-5p as a ceRNA and upregulate the expression of high-mobility group box 1 (HMGB1) in the spinal cord, promoting the development of CNP (Zhao et al., 2016). lncRNA CRNDE can upregulate the expression of IL-6 receptors in chronic pain by interacting with miR-136 (Zhang et al., 2019). In addition, lncRNA Linc01119 can interact with embryonic lethal abnormal version-like RNA-binding protein 1 (ELAVL1), upregulate the expression of brain-derived neurotrophic factor (BDNF) at the mRNA and protein levels, and induce chronic pain in the spinal cord and DRG (Zhang L. et al., 2021). In summary, lncRNAs can interact with miRNA or RNA-associated proteins and regulate the different downstream mechanisms involved in chronic pain.

**FIGURE 1:**
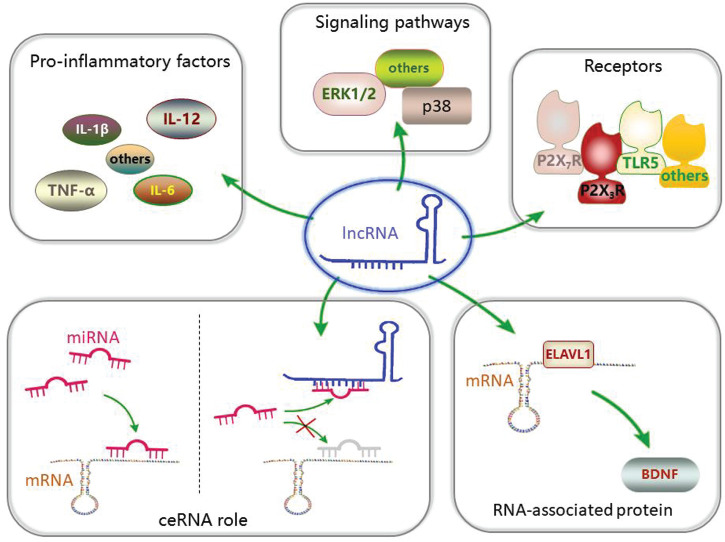
Long non-coding RNA (lncRNAs) and their mechanism in pain transmission: lncRNAs can participate in pain transmission through various mechanisms.

In addition, some lncRNAs have been reported to mediate the activation of signaling pathways (Ren et al., 2020) and participate in the development of chronic pain. lncRNA LOC100911498 small interfering RNA (siRNA) treatment can decrease the phosphorylation of the p38 pathway in the spinal cord induced by chronic pain (Tang et al., 2021). Another study suggested that activation of the ERK1/2 pathway in the DRG is regulated by lncRNA uc.48+ (Wang et al., 2016). p38 and ERK1/2 can participate in the development of chronic pain (Lin et al., 2014; Qian et al., 2019). In addition, P2X_3_ and P2X_7_ receptors have been found to be regulated by lncRNAs (Seino et al., 2006; Peng H. et al., 2017). The two receptors play a role in the development of chronic pain (Wu et al., 2021; Xia et al., 2021). Furthermore, the levels of pro-inflammatory factors, such as IL-1β, IL-6, IL-12, and TNF-α (Xia et al., 2018; Li Z. et al., 2020; Pan et al., 2020), have been found to change in chronic pain after lncRNA downregulation. Neuroinflammation plays a significant role in chronic pain. Thus, the effect of lncRNAs on the development of chronic pain may involve various mechanisms (Figure 1).

## lncRNAs and Chronic Neuropathic Pain

Chronic neuropathic pain (CNP), a major public health concern worldwide, affects the quality of life of 6.9–10% of the general population (van Hecke et al., 2014). CNP is characterized by spontaneous ongoing or evoked by sensory stimuli (hyperalgesia and allodynia). It is mainly observed in peripheral nerve lesions [diabetic neuropathy, peripheral nerve injury (PNI), and trigeminal neuralgia (TN)] or central nerve lesions (SCI) (Scholz et al., 2019). Various animal models of peripheral neuropathic pain and central neuropathic pain have been established to explore the mechanisms underlying the development of CNP (Tian et al., 2020; Zhang P. et al., 2021). However, the treatment of CNP remains a major challenge. Recent accumulating evidence has shown that lncRNAs are related to the development of peripheral neuropathic pain and central neuropathic pain (Liu et al., 2018; Sun et al., 2018; Tian et al., 2020; Xu et al., 2020).

### lncRNAs and Peripheral Neuropathic Pain

#### lncRNAs and Peripheral Nerve Injury

**FIGURE 2:**
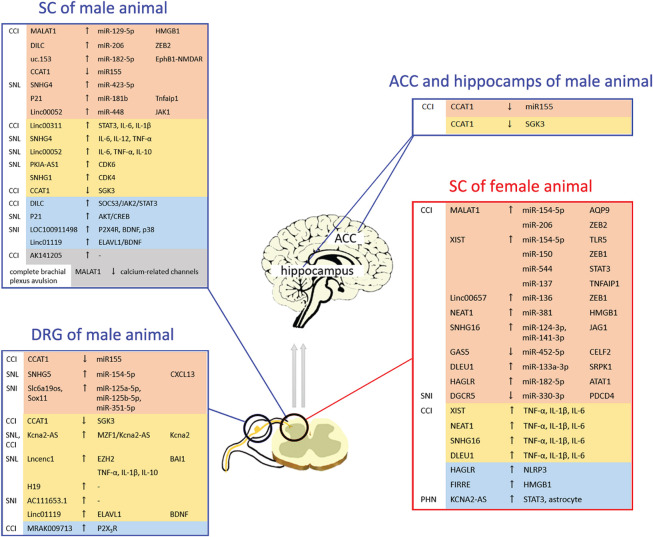
Distribution of deregulated long non-coding RNAs (lncRNAs) with the respective associated mechanisms in peripheral nerve injury (PNI): lncRNAs can participate in the development of PNI-induced chronic neuropathic pain (CNP) through various mechanisms. The same lncRNA displays different expressions in different models or issues. Most lncRNAs can play an important role in the development of PNI-induced CNP in both female and male models. ACC, anterior cingulate cortex; DRG, dorsal root ganglion; SC, spinal cord.

Peripheral nerve injury, which induces CNP, is a common clinical cause of peripheral nerve lesions. PNI can cause excitability of the primary sensory ganglia or the spinal cord in the nervous system (Tsuda et al., 2009), which plays a role in pain-signaling transmission. Most animal models, such as those of chronic constriction injury (CCI), spinal nerve ligation (SNL), and spared sciatic nerve injury (SNI), have been used to investigate the relationship between lncRNAs and CNP in the nervous system (Table 1 and Figure 2). Zhao et al. (2013) were the first to show that the expression of a new native lncRNA was upregulated in mammalian DRG neurons of SNL and CCI model mice. Since the sequence of this lncRNA was found to be complementary to that of KCNA2 RNA, the researchers named it as KCNA antisense (KCNA2-AS). KCNA2-AS was identified to trigger the downregulation of KCNA2 in the DRG and participate in the development of neuropathic pain by using KCNA2-AS siRNA, indicating the important role of lncRNAs in CNP development. The following studies were performed to explore the roles and mechanisms of lncRNAs in the development of PNI-induced CNP. lncRNAs, such as MALAT1, DILC, FIRRE, XIST, H19, and DGCR5 in the spinal cord, have been found to have a continuous effect on CNP (Wei et al., 2018; Peng et al., 2019; Li K. et al., 2020; Liu et al., 2020; Wu et al., 2020; Wen et al., 2021). lncRNAs, such as H19, SNHG5, and MRAK009713 in DRG, have also been identified to play important roles in the development of CNP (Li et al., 2017; Chen et al., 2020; Wen et al., 2020). Since numerous lncRNAs are involved, we have summarized the following points:

**TABLE 1:** lncRNAs and peripheral nerve injury.

**Model**	**lncRNAs**	**Distribution**	**Expression**	**Mechanism**	**References**
CCI	MALAT1	SC of female rat	↑	MALAT1/miR-154-5p/AQP9 axis	Wu et al., 2020
		SC of female rat	↑	MALAT1/miR-206/ZEB2 axis	Chen Z.L. et al., 2019
		SC of male rat	↑	MALAT1/miR-129-5p/HMGB1 axis	Ma et al., 2020
	DILC	SC of male rat	↑	SOCS3/JAK2/STAT3 pathway	Liu et al., 2020
	FIRRE	SC of female mouse	↑	HMGB1	Wen et al., 2021
	CRNDE	SC of rat	↑	CRNDE/miR-136/IL6R axis, IL-1, IL-6, IL-10, TNF-α	Zhang et al., 2019
	XIST	SC of female rat	↑	XIST/miR-154-5p/TLR5 axis	Wei et al., 2018
		SC of female rat	↑	XIST/miR-150/ZEB1 axis	Yan et al., 2018
		SC of female rat	↑	XIST/miR-544/STAT3 axis, TNF-α, IL-1β, IL-6	Jin et al., 2018
		SC of female rat	↑	XIST/miR-137/TNFAIP1 axis	Zhao et al., 2018
	Linc00657	SC of female rat	↑	Linc00657/miR-136/ZEB1 axis	Shen et al., 2019
	NEAT1	SC of female rat	↑	NEAT1/miR-381/HMGB1 axis, IL-6, IL-1β, TNF-α	Xia et al., 2018
	uc.153	SC of male mouse	↑	uc.153/miR-182-5p/EphB1-NMDA receptors	Zhang C. et al., 2020
	Linc00311, AK141205	SC of male rat	↑	STAT3, IL-6, IL-1β	Pang et al., 2020
	SNHG16	SC of female rat	↑	SNHG16/miR-124-3p, miR-141-3p/JAG1 axis, IL- 6, TNF-α, IL-1β	Li H. et al., 2020
	GAS5	SC of female rat	↓	GAS5/miR-452-5p/CELF2 axis	Tian et al., 2020
	DLEU1	SC of female rat	↑	DLEU1/miR-133a-3p/SRPK1 axis, IL-6, TNF-α, IL-1β	Li Z. et al., 2020
	H19	SC of rat	↑	H19/miR-196a-5p/CDK5 axis, p-CREB	Li K. et al., 2020
	HAGLR	SC of female rat	↑	HAGLR/miR-182-5p/ATAT1 axis, NLRP3	Zhang Q. et al., 2021
	CCAT1	Hippocampus, SC, DRG of male rat	↓	miR155, SGK3	Dou et al., 2017
	MRAK009713	DRG of male rat	↑	P2X_3_ receptor	Li et al., 2017
SNL, CCI	Kcna2-AS	DRG neuron of male rat	↑	MZF1/Kcna2-AS/Kcna2	Zhao et al., 2013
SNL	PKIA-AS1	SC of male rat	↑	CDK6	Hu et al., 2019
	SNHG1	SC of male rat	↑	CDK4	Zhang J.Y. et al., 2020
	SNHG4	SC of male rat	↑	miR-423-5p, IL-6, IL-12, TNF-α	Pan et al., 2020
	SNHG5	L5 DRG of male mouse	↑	SNHG5/miR-154-5p/CXCL13 axis	Chen et al., 2020
	P21	SC of male rat	↑	P21/miR-181b/Tnfaip1, AKT/CREB axis	Liu et al., 2021
	Linc00052	SC of male rat	↑	Linc00052/miR-448/JAK1 axis, IL-6, TNF-α	Wang L. et al., 2020
	H19	DRG of male mouse	↑	Unknown	Wen et al., 2020
	Lncenc1	DRG of male mouse	↑	Lncenc1/EZH2/BAI1 TNF-α, IL-1β, IL-10	Zhang Z. et al., 2021
SNI	AC111653.1	DRG of male rat	↑	Unknown	Mao et al., 2018
	DGCR5	SC of female rat	↓	DGCR5/miR-330-3p/PDCD4 axis	Peng et al., 2019
	LOC100911498	L4, L5 SC of male rat	↑	P2X_4_R, BDNF, p38	Tang et al., 2021
	Slc6a19os, Sox11	L3-L5 DRG of male mouse	↑	miR-125a-5p, miR-125b-5p, miR-351-5p	Chen et al., 2021
	Linc01119	L4-L5 SC and DRGs of male rats	↑	Linc01119/ELAVL1/BDNF axis	Zhang L. et al., 2021
Complete brachial plexus avulsion	MALAT1	cytoplasm of neurons in male rat C5-T1 SC	↓	Unknown	Meng et al., 2019
PHN	Kcna2-AS	L4, L5 SC of female rat	↑	STAT3, astrocyte	Kong et al., 2020

*CCI, chronic constriction injury, SNL, spinal nerve ligation; SNI, spared sciatic nerve injury; SCI, spinal cord injury; SC, spinal cord; DRG, dorsal root ganglion; PHN, postherpetic neuralgia; ↑, upregulated expression; ↓, downregulated expression.*

(1) Most lncRNAs interacted with miRNAs, and MALAT1 and XIST were the most common among these lncRNAs. miRNA downregulation triggered by these lncRNAs could influence the downstream mechanism and induce mechanical and cold hypersensitivity and the symptoms of PNI-associated CNP. In addition, other mechanisms of lncRNAs have been investigated in PNI models. DILC, Linc00311, AK141205, and KCNA2-AS have been reported to participate in CNP by regulating the JAK/STAT3-signaling pathway (Mao et al., 2018; Kong et al., 2020; Liu et al., 2020). MRAK009713-mediated CNP development is involved in P2X_3_ receptor activation (Li et al., 2017). Cyclin-dependent kinases 4 and 6 (CDK4 and CDK6) have been found to be regulated by lncRNA SNHG1 and PKIA-AS1, respectively (Hu et al., 2019; Zhang J.Y. et al., 2020). Notably, changes in the levels of pro-inflammatory cytokines (IL-1β, IL-6, and TNF-α) have been found in most PNI models (Li Z. et al., 2020; Pan et al., 2020), indicating that lncRNA-mediated CNP development may be involved in neuroinflammation.
(2) The same issue could express various lncRNAs, which may be different expression levels or play opposite effect on the PNI model. In the spinal cord of CCI rats, DLEU1 expression was upregulated (Li Z. et al., 2020), whereas GAS5 expression was downregulated (Tian et al., 2020). Thus, the two lncRNAs played opposite roles in the development of PNI-induced CNP. In addition, the expression of the same lncRNA in different conditions or models may display opposite changes. MALAT1 expression was increased in the L4-L6 spinal cord of male CCI rats (Ma et al., 2020), while its expression was reduced in the C5-T1 spinal cord of male complete brachial plexus avulsion rats (Meng et al., 2019).
(3) Owing to the sex difference in pain sensitivity (Fullerton et al., 2018), clinical and experimental findings have suggested that women are more sensitive to pain than men (Fillingim et al., 2009). lncRNA XIST, which mediates X-chromosome inactivation or reactivation in female cells (Vacca et al., 2016), has been found to play an important role in female PNI models (Jin et al., 2018; Wei et al., 2018; Yan et al., 2018; Zhao et al., 2018). However, most lncRNAs exert their effect on all PNI models, regardless of sex, indicating that most lncRNAs can play an important role in the development of PNI-induced CNP in both female and male models.
(4) Many studies have paid attention to the effect of lncRNAs on spinal cord and DRG. However, various specific brain regions, such as hippocampus, periaqueductal gray (PAG), anterior cingulate cortex (ACC), can also exert their effect on the development of chronic pain (Bliss et al., 2016; Ong et al., 2019). Dou et al. (2017) found the decrease of the lncRNA CCAT1 level in hippocampus and ACC of the CCI model. Overexpression of CCAT1 could alleviate CNP and inhibit the increased miR-155. As a role of ceRNA, CCAT1 could inhibit miRNA expression, and the researcher further identified the role of serum and glucocorticoid-regulated protein kinase 3 (SGK3) in CCAT1-mediated miR-155 expression and CCAT1-induced CNP. These results indicated the significant role of lncRNA in hippocampus and ACC. However, the effect of lncRNAs on specific brain regions needs to be explored in the future.

#### lncRNAs and Diabetic Neuropathic Pain

Diabetic neuropathic pain (DNP), a painful diabetic peripheral neuropathy, is one of the most common types of neuropathic pain (de Vos et al., 2014), and it commonly manifests as allodynia, hyperalgesia, or spontaneous pain (Wang et al., 2014). Approximately, 40–50% of patients with diabetes experience DNP (Schreiber et al., 2015), whereas effective therapies for DNP remain elusive. Recently, genome-wide expression patterns of lncRNAs have been identified, and RT-qPCR validated the dysregulation of lncRNAs in the spinal cord of DNP mice (Du et al., 2019). Bioinformatics analysis results have shown that these lncRNA-related genes are involved in calcium ion transport, which participates in neuropathic pain development (Baba et al., 2016). However, the speculation that lncRNA exerts an effect on the ion channel in DNP needs to be further explored (Table 2).

**TABLE 2:** lncRNAs and diabetic neuropathic pain.

**Model**	**lncRNAs**	**Distribution**	**Expression**	**Mechanism**	**References**
DNP	BC168687	DRG of male rat	↑	TRPV1, ERK1/2, p38, TNF-α, IL-1β	Liu et al., 2018
		DRG of male rat	↑	P2X_7_R, NO	Liu et al., 2017
	NONRATT021972	DRG of rat	↑	P2X_7_R, TNF-α, astrocyte	Liu et al., 2016
		DRG of male rat	↑	P2X_3_R, ERK1/2	Peng H. et al., 2017
		Blood sample of male rat	↑	TNF-α	Yu et al., 2017
	uc.48+	DRG of male rat	↑	P2X_3_R, ERK1/2	Wang et al., 2016
		SC of male rat	↑	CGRP, ERK1/2, p38	Xiong et al., 2017

*DNP, diabetic neuropathic pain; ↑, upregulated expression.*

Liu et al. (2018) identified the role of lncRNAs in DNP by regulating transient receptor potential vanilloid type 1 (TRPV1) activation in the rat DRG. Using western blot analysis, they found that high TRPV1 receptor expression in DRG neurons of DNP rats could be substantially decreased by lncRNA BC168687 siRNA, which could alleviate TRPV1-mediated diabetic neuropathic pain (Zhang B.Y. et al., 2020), indicating that lncRNA BC168687 may regulate the ion channel of DRG neurons and participate in the development of DNP. In addition, Liu et al. (2018) found that P2X_7_ receptor expression was downregulated after BC168687 siRNA treatment. The P2X_7_ receptor is mainly expressed in satellite glial cells (SGCs) (Chen et al., 2012; Puchałowicz et al., 2015), which tightly enwrap the DRG (Costa and Moreira Neto, 2015). Previous studies have suggested that SGC P2X_7_ receptors play an important role in neuropathic pain (Kuan and Shyu, 2016; Bernier et al., 2018). Liu et al. (2017) found that treatment with BC168687 siRNA decreased the serum level of oxidative injury factors (e.g., NO) released by SGCs in a DNP model. NO can strengthen the sensitivity of neurons to noxious stimulation in the DRG (Thippeswamy et al., 2005). NO has been reported to be involved in the development of neuropathic pain (Rondón et al., 2018). Thus, BC168687 may promote interaction with neurons and glia in the DRG during DNP. These data indicate that lncRNA BC168687 in DRG may participate in the development of DNP by regulating the activation of both neurons and glia.

Long non-coding RNA NONRATT021972 has also been validated to play an important role in the development of DNP (Liu et al., 2016). Using lncRNA siRNA, P2X_7_ antagonist, and electrophysiological recordings of neurons, this lncRNA was found to regulate P2X_7_ receptor expression in the SGCs of the DRG during DNP. Peng H. et al. (2017) explored the direct effect of this lncRNA on DRG neurons. NONRATT021972 siRNA inhibited the expression and activation of the P2X_3_ receptor and its downstream ERK1/2-signaling pathway in neurons and relieved DNP. The ERK1/2-signaling pathway is involved in neuropathic pain transmission (Seino et al., 2006). These results indicate that lncRNA NONRATT021972 in the DRG may participate in the development of DNP by regulating the activation of both neurons and glia.

Similarly, the P2X_3_ receptor and ERK1/2-signaling pathway in the DRG are regulated by another lncRNA uc.48+ (Wang et al., 2016). In addition, lncRNA uc.48+ siRNA can significantly suppress the expression of calcitonin gene-related peptide (CGRP), IL-1β, and TNF-α in the spinal cord (Xiong et al., 2017). The expression of CGRP, IL-1β, and TNF-α in the spinal cord may contribute to pain responses (Brown et al., 2008; Hansen et al., 2016). Thus, lncRNA uc.48+ may participate in the development of DNP by regulating the expression of the three factors in the spinal cord. The findings from the aforementioned studies suggest a role for lncRNA uc.48+ in the progression of DNP and provide various lines of evidence to explain the lncRNA-mediated mechanisms underlying the development of DNP.

#### lncRNAs and Trigeminal Neuralgia

Trigeminal neuralgia is a common type of neuropathic pain, and many treatments for TN, including medical therapy and microvascular decompression, have been found to be ineffective (Bick and Eskandar, 2017). Recently, lncRNA Gm14461 expression has been found to be increased in the trigeminal ganglia (TG) of TN mice (Xu et al., 2020). The Gm14461 knockdown increased the mechanical withdrawal threshold of TN mice, indicating that Gm14461 may play a regulatory role in mechanical hyperalgesia in TN mice. Western blot analysis results suggested that the Gm14461 knockdown could downregulate the expression of CGRP and P2X_3__/__7_ receptors at the protein level in TN mice. The three proteins are reported to participate in the development of neuropathic pain (Hansen et al., 2016; Wu et al., 2021; Xia et al., 2021). Moreover, Gm14461 upregulates the expression of TNF-α, IL-1β, and IL-6 (Xu et al., 2020). Another lncRNA uc.48+ interacts with the P2X_7_ receptor and promotes the expression of the P2X_7_ receptor in TG (Xiong et al., 2019). Western blot analysis results suggests that the ERK-signaling pathway may be involved in this interaction between uc.48+ and P2X_7_ receptor. These findings suggest that lncRNAs may play an important role in the development of TN through various mechanisms.

### lncRNAs and Central Neuropathic Pain Associated With Spinal Cord Injury

Chronic neuropathic pain is one of the most common complications of SCI that severely influences the quality of life of patients with SCI (Bouhassira et al., 2008). A bioinformatics analysis was performed to determine the dysregulation of lncRNA expression associated with pain transmission in blood samples from patients with SCI (Zhao et al., 2021). Two lncRNAs (Linc01119 and Linc02447) involved in the pain pathway indicated that lncRNA-mediated pain transmission may play a role in the development of SCI-induced CNP. Xian et al. (2021) confirmed the role of lncRNAs in the spinal cord of the CNP model. lncRNA NEAT1 expression was increased in the spinal cord of SCI rats, and NEAT1 inhibition alleviated SCI-induced CNP. miR-128-3p was downregulated by NEAT1 overexpression, as it played the role of its ceRNA, and the levels of AQP4, IL-6, IL-1β, and TNFα were increased after miR-128-3p inhibition. Another study suggested that upregulated lncRNA PVT1 could alleviate SCI-induced CNP by targeting the miR-186-5p/CXCL13/CXCR5 axis (Zhang P. et al., 2021). CXCL13, CXCR5, and AQP4 are vital regulators of the inflammatory response in the nervous system (Liang et al., 2016; Bu et al., 2019). Thus, these two studies indicated the role and the mechanism of lncRNAs in the development of SCI-induced CNP, including their interaction with miRNAs or indirect regulation of the inflammatory response.

## lncRNAs and Complex Regional Pain Syndrome-Induced Inflammatory Pain

Complex regional pain syndrome (CRPS) is a chronic pain disorder characterized by intense pain, inflammation, and altered autonomic function (de Mos et al., 2007). The mechanism underlying the development of CRPS remains unclear (Birklein and Schlereth, 2015). Since women are about four times more likely than men to develop CRPS (Schwartzman et al., 2009), Shenoda et al. (2018) investigated the role of lncRNA XIST in the development of CRPS. XIST promotes and maintains X-chromosome inactivation (Wang et al., 2021), which refers to the random selection and transcriptional silencing of one of the two X-chromosomes in females, indicating the association of its effect with sex differences. RT-qPCR analysis results suggested that the expression of XIST was increased, and the upstream expression of miR-34a was decreased in the blood samples of patients with CRPS (Shenoda et al., 2018). As a role of a ceRNA, XIST in blood was identified to be directly regulated by miR-34a in a complete Freund’s adjuvant (CFA)-induced inflammatory pain model. The pro-inflammatory transcription factor, Yin-Yang 1 (YY1), was found to participate in miR-34a-mediated XIST expression. Thus, miRNA-mediated downregulation of XIST expression in the blood may be a potential strategy for relieving CRPS-induced inflammatory pain. Another study found that XIST expression in the DRG was increased in a CFA-induced inflammatory pain model, and the XIST knockdown inhibited activated the Nav1.7 channel and levels of IL-6 and TNF-α in the DRG and attenuated inflammatory pain (Sun et al., 2018). These studies indicate that XIST is regulated by miRNAs and mediates the release of pro-inflammatory factors, participating in the development of inflammatory pain, demonstrating a new mechanism underlying inflammatory pain. However, the mechanism underlying CRPS-induced inflammatory pain in male patients needs to be further explored.

## lncRNAs and Osteoarthritis-Induced Inflammatory Pain

Osteoarthritis is one of the most common forms of arthritis (Sellam and Berenbaum, 2010). Its clinical manifestations include joint swelling, synovitis, and inflammatory pain, which cause pain to the patient. Many studies have indicated the regulatory role of lncRNAs in the inflammatory process of OA. Many lncRNAs can attenuate OA through the interaction between lncRNAs and miRNAs (Xie et al., 2020), MAPK pathway (Xiao et al., 2019), and pro-inflammatory factors (Li et al., 2018a). However, the role of lncRNAs in OA-induced inflammatory pain remains unclear. This review focused on lncRNAs involved in this type of pain. Similar to lncRNAs in the PNI model, various lncRNAs may be differentially expressed and exert opposite effects in the pathogenesis of OA (Abbasifard et al., 2020; Xie et al., 2020). Li et al. (2018b) first found that the levels of lncRNA MEG3 increased in the articular tissue of an OA model after treatment with methylene blue, which improved pain sensitivity and reduced inflammatory pain in the OA model. MEG3 has been reported to play a protective role in chondrocytes against IL-1β-induced inflammation in an OA model (Huang et al., 2021). IL-1β, IL-6, and TNFα levels were decreased in a methylene blue-treated OA model, and MEG3 siRNA increased the expression of IL-1β, IL-6, and TNF-α reduced, following methylene blue treatment (Li et al., 2018b), indicating that lncRNA MEG3 may alleviate OA-induced pain by regulating inflammation. Subsequently, research was performed to investigate the effect of lncRNAs on the nervous system (Yang et al., 2021). Umbilical cord blood mesenchymal stem cells, which can release exosomes containing lncRNA H19, were intravenously, intracavitary, or intrathecally administered to an OA model, and all three types of administrations improved the pain sensitization of advanced OA. RT-qPCR analysis results suggested that serum IL-1α, IL-2, IL-6, and TNF-αlevels were decreased by treatment with exo-lncRNA H19. In addition, activation of the ERK-signaling pathway in the spinal dorsal horn was inhibited by exo-lncRNA H19. These results indicate that lncRNAs may regulate the development of OA-induced pain *via* different mechanisms. Notably, different lncRNAs may play opposite roles in the inflammatory process of OA (Abbasifard et al., 2020; Xie et al., 2020); a similar phenomenon may appear in the development of OA-induced pain. The roles and mechanisms of different lncRNAs need to be validated.

## lncRNA and Chronic Cancer-Related Pain

**FIGURE 3:**
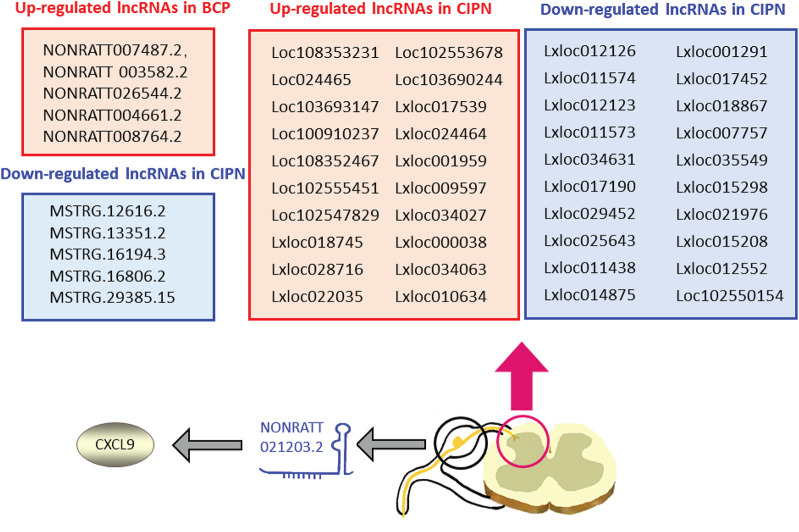
Expression changes and mechanisms of long non-coding RNAs (lncRNAs) in CCRP: many lncRNAs are dysregulated by tumor metastasis and chemotherapy treatment. lncRNA NONRATT021203.2 can exert its effect on BCP by mediating CXCL9 release. BCP, bone cancer pain; CCRP, chronic cancer-related pain; CIPN, chemotherapy-induced peripheral neuropathy.

Chronic cancer-related pain, another type of chronic pain, similarly deteriorates the quality of life of patients. It can be caused by cancer itself (primary tumor or metastases) or by its treatment (surgery, chemotherapy, and radiotherapy) (Bennett et al., 2019). CCRP is characterized by symptoms of syndromes, including neuropathic and musculoskeletal pain (Treede et al., 2019). Many lncRNAs have been found to play a significant role in cancer, cancer metastasis, and cancer-associated treatment (Li et al., 2016, 2021; Peng W.X. et al., 2017). Recently, accumulating evidence has shown that lncRNAs are related to the development of CCRP (Figure 3).

### lncRNAs and Cancer-Induced Pain

More than 50% of patients with cancer experience cancer-induced pain (CIP) (van den Beuken-van et al., 2016). Bone cancer pain (BCP) is the most common type of CIP and is mainly caused by metastatic tumors (Bennett et al., 2019). Many studies have focused on the role of lncRNAs in tumor metastasis (Weidle et al., 2017). This review focuses on the latest research findings on the effect of lncRNAs on CIP and identifies the roles of lncRNAs in metastatic tumor-induced pain. Transcriptome sequencing and RT-qPCR validated the change in the expression of 10 lncRNAs (five upregulated and five downregulated) in the ipsilateral lumbar spinal cord in a rat BCP model (Hou et al., 2020). Gene Ontology (GO) and Kyoto Encyclopedia of Genes and Genomes (KEGG) analysis of the dysregulated lncRNAs (NONRATT007487.2, NONRATT 003582.2, NONRATT026544.2, NONRATT004661.2, NONRATT008764.2, MSTRG.12616.2, MSTRG.13351.2, MSTRG.16194.3, MSTRG.16806.2, and MSTRG.29385.15) indicated that they were mainly involved in inflammatory and immunological responses. Inflammation in the nervous system has been reported to play an important role in BCP, and inhibiting this response could significantly attenuate BCP (Song et al., 2015; Chen S.P. et al., 2019), indicating the potential role of lncRNAs in the development of BCP. Another study further confirmed the link between lncRNAs and neuroinflammation in a BCP model (Sun et al., 2020). The researchers relieved hyperalgesia in BCP rats by treatment with lncRNA NONRATT021203.2 siRNA. In addition, the increased expression of C-X-C motif chemokine ligand 9 (CXCL9) in the DRG was inhibited by this siRNA. CXCL9 has been reported to play a pro-neuroinflammation role in the nervous system (Koper et al., 2018), and inhibiting CXCL9 expression could relieve hyperalgesia in BCP rats (Sun et al., 2020), indicating that NONRATT021203.2 could target CXCL9 and result in CIP in the BCP model. The findings from the two studies indicate that the lncRNA-neuroinflammation axis may be a potential target for the treatment of CIP.

### lncRNA and Chemotherapy-Induced Pain

Chemotherapy-induced peripheral neuropathy is a neurotoxic adverse effect of many chemotherapeutic agents (Banach et al., 2017). Chronic pain is a major symptom of chemotherapy-induced peripheral neuropathy (CIPN) (Brewer et al., 2016). The mechanism underlying chemotherapy-induced pain remains unclear, and many medical treatments are usually insufficient for pain management (Sisignano et al., 2014). In a recent study, RNA sequencing (RNA-Seq) and bioinformatics analysis have been performed to explore lncRNA expression profiles in the spinal cord dorsal horn of rats treated with paclitaxel (Li et al., 2021), one of the most commonly used chemotherapeutic agents (Mody et al., 2016). These results suggest that dysregulated lncRNAs were primarily involved in the neurotrophin-signaling pathway. Neurotrophin signaling could result in the recruitment of signaling proteins (Scott-Solomon and Kuruvilla, 2018), which activate downstream intracellular-signaling pathways, including the ERK1/2 and NF-κB pathways. ERK1/2 and NF-κB signaling have been found to participate in paclitaxel-induced peripheral neuropathy (Wang G. J. et al., 2020; Zhao et al., 2020). These two signaling pathways have been identified downstream of lncRNAs (Peng H. et al., 2017; Zhao et al., 2018). This study indicated that lncRNAs may play an important role in the process of chemotherapy-induced pain by mediating the two signaling pathways; however, this needs to be further validated.

## Conclusion

In recent years, an increasing number of studies have addressed the change in expression of lncRNAs in humans with chronic pain and preclinical pain models. The vital role of lncRNAs in chronic pain, including CNP, inflammatory pain, and CCRP, has been identified. These lncRNAs can participate in the development of chronic pain by interacting with miRNAs, regulating pro-inflammatory cytokine levels, and mediating signaling pathways. However, the regulatory effects of lncRNAs may be contradictory in different models or different issues. Some lncRNAs, such as XIST, are associated with sex-related differences. Thus, it is necessary to take these factors into account while exploring strategies for alleviating chronic pain. In addition, the same lncRNA could exert its effect on different types of chronic pain, indicating the existence of a similar mechanism underlying the development of different types of pain. Although lncRNA-based clinical agents for chronic pain have not been clearly determined, this preclinical exploration of the mechanism may provide novel and evidential insights for exploring effective strategies for lncRNA-based treatments for chronic pain. However, the clinical efficacy and risks involved in lncRNA therapy need to be systematically evaluated.

## Author Contributions

ZL wrote the first draft of the manuscript. XL and WJ accessed the data. QX and ZHL contributed to the manuscript revision. All authors contributed to the conception and design of the study, contributed to the article, and approved the submitted version.

## Conflict of Interest

The authors declare that the research was conducted in the absence of any commercial or financial relationships that could be construed as a potential conflict of interest.

## Publisher’s Note

All claims expressed in this article are solely those of the authors and do not necessarily represent those of their affiliated organizations, or those of the publisher, the editors and the reviewers. Any product that may be evaluated in this article, or claim that may be made by its manufacturer, is not guaranteed or endorsed by the publisher.

## Abbreviations

AQP4: Aquaporin 4
BCP: bone cancer pain
BDNF: brain-derived neurotrophic factor
CCRP: chronic cancer-related pain
CCI: chronic constriction injury
CDK: cyclin-dependent kinase
ceRNA: competitive endogenous RNA
CFA: Complete Freund’s Adjuvant
CGRP: calcitonin gene-related peptide
CIP: cancer-induced pain
CIPN: chemotherapy-induced peripheral neuropathy
CNP: chronic neuropathic pain
CRNDE: colorectal neoplasia differentially expressed
CRPS: complex regional pain syndrome
CXCL13: chemokine ligand 13
CXCL9: chemokine ligand 9
CXCR5: chemokine receptor 5
DGCR5: DiGeorge syndrome critical region gene 5
DILC: downregulated in liver cancer stem cells
DLEU1: deleted in lymphocytic leukemia 1
DNP: diabetic neuropathic pain
DRG: dorsal root ganglion
ELAVL1: embryonic lethal abnormal version-like RNA-binding protein 1
ERK 1/2: extracellular regulated protein kinases 1/2
exo-lncRNA H19: exosome containing lncRNA H19
FIRRE: functional intergenic repeating RNA element
GAS5: growth-arrest-specific 5
GO: Gene ontology
HMGB1: high-mobility group box 1
IL-1 β: Interleukin-1 β
IL-6: Interleukin-6
IL-12: Interleukin-12
JAK: Janus kinase
KCNA2-AS: KCNA antisense RNA
KEGG: Kyoto Encyclopedia of Genes and Genomes
lncRNAs: long non-coding RNAs
MALAT1: Metastasis-associated lung adenocarcinoma transcript (MALAT)1
MAPK: mitogen-activated protein kinases
MEG3: maternally expressed gene 3
miR: miRNA
NEAT1: nuclear paraspeckle assembly transcript 1
NF- κ B: nuclear factor-kappaB
NO: nitric oxide
OA: osteoarthritis
P2X_3_R: P2X_3_ receptor
P2X_7_R: P2X_7_ receptor
PNI: peripheral nerve injury
PVT1: plasmacytoma variant translocation 1
SC: spinal cord
SCI: spinal cord injury
SGCs: satellite glial cells
siRNA: small interference RNA
SNHG1: small nucleolar RNA host gene 1
SNHG5: small nucleolar RNA host gene 5
SNI: spared sciatic nerve injury
SNL: spinal nerve ligation
STAT3: signal transducer and activator of transcription 3
TG: trigeminal ganglia
TN: trigeminal neuralgia
TNF- α: tumor necrosis factor
TRPV1: transient receptor potential vanilloid type 1
UCBMSCs: umbilical cord blood mesenchymal stem cells
XIST: X-inactive specific transcript
YY1: Yin-Yang 1.
